# Mir-21 Mediates the Inhibitory Effect of Ang (1–7) on AngII-induced NLRP3 Inflammasome Activation by Targeting Spry1 in lung fibroblasts

**DOI:** 10.1038/s41598-017-13305-3

**Published:** 2017-10-30

**Authors:** Na-Na Sun, Chang-Hui Yu, Miao-Xia Pan, Yue Zhang, Bo-Jun Zheng, Qian-Jie Yang, Ze-Mao Zheng, Ying Meng

**Affiliations:** Department of Respiratory Diseases, Nanfang Hospital, Southern Medical University, Guangzhou, China

## Abstract

MicroRNA-21 (mir-21) induced by angiotensin II (AngII) plays a vital role in the development of pulmonary fibrosis, and the NLRP3 inflammasome is known to be involved in fibrogenesis. However, whether there is a link between mir-21 and the NLRP3 inflammasome in pulmonary fibrosis is unknown. Angiotensin-converting enzyme 2/angiotensin(1–7) [ACE2/Ang(1–7)] has been shown to attenuate AngII-induced pulmonary fibrosis, but it is not clear whether ACE2/Ang(1–7) protects against pulmonary fibrosis by inhibiting AngII-induced mir-21 expression. This study’s aim was to investigate whether mir-21 activates the NLRP3 inflammasome and mediates the different effects of AngII and ACE2/Ang(1–7) on lung fibroblast apoptosis and collagen synthesis. *In vivo*, AngII exacerbated bleomycin (BLM)-induced lung fibrosis in rats, and elevated mir-21 and the NLRP3 inflammasome. In contrast, ACE2/Ang(1–7) attenuated BLM-induced lung fibrosis, and decreased mir-21 and the NLRP3 inflammasome. *In vitro*, AngII activated the NLRP3 inflammasome by up-regulating mir-21, and ACE2/Ang(1–7) inhibited NLRP3 inflammasome activation by down-regulating AngII-induced mir-21. Over-expression of mir-21 activated the NLRP3 inflammasome via the ERK/NF-κB pathway by targeting Spry1, resulting in apoptosis resistance and collagen synthesis in lung fibroblasts. These results indicate that mir-21 mediates the inhibitory effect of ACE2/Ang(1–7) on AngII-induced activation of the NLRP3 inflammasome by targeting Spry1 in lung fibroblasts.

## Introduction

Pulmonary fibrosis is a progressive and fatal lung disease. The disease is characterized by progressive scaring of the lung tissue accompanied by fibroblast proliferation, excessive accumulation of matrix proteins, and an abnormal alveolar structure, all leading to loss of lung function and, ultimately, respiratory failure^1^.

MicroRNAs are non-coding RNA molecules that regulate gene expression at a transcriptional and post-transcriptional level^2^ in biological and pathological processes^3^. Both clinical and experimental animal studies have revealed that aberrant expression of miRNAs is associated with the development of fibrotic diseases^4–6^. Recent studies have shown that microRNA-21 (mir-21) is increased in the lung of bleomycin (BLM)-treated mice as well as in patients with idiopathic pulmonary fibrosis (IPF)^2^, and that it mediates lung fibroblast activation and fibrosis^7^. Spry1, a direct target of mir-21, augments extracellular signal-regulated kinase (ERK) activity in cardiac fibroblasts^8^ and hematopoietic stem cells (HSCs)^9^. However, it has not been determined whether mir-21 mediates pulmonary fibrosis via the ERK signaling pathway by targeting Spry1.

Increasing evidence suggests that the NLRP3 inflamasome can drive fibrotic responses^10,11^. It can directly promote collagen synthesis, leading to collagen deposition in the lung, liver, heart, and skin^12^. The NLRP3 inflammasome is a multimolecular protein complex responsible for caspase-1-drived activation of the pro-inflammatory cytokine interleukin (IL)−1β^13^. Biologically active IL-1β is essential for regulating the host response that can lead to excessive inflammation and fibrosis in the lung^14^. In a review of inflammasomes in liver diseases, Szabo & Csak^15^ cite data showing that IL-1Ra administration attenuates dimethylnitrosamine (DMN)-induced liver cirrhosis, and that IL-1R-deficient mice are protected from thioacetamide (TAA)-induced fibrogenesis. Therefore, the NLRP3 inflammasome/IL-1β secretion axis is a promising therapeutic target for the prevention and treatment of lung fibrosis.

The NLRP3 inflammasome can be activated by diverse stimuli and a 2-signal model has been proposed for NLRP3 inflammasome activation. Signal 1 is provided by microbial molecules or endogenous cytokines, in which activation of NF-κB (nuclear factor kappa-light-chain-enhancer of activated B cells**)** leads to up-regulation of NLRP3 and pro-IL-1β^16^. Ling *et al*.^17^ and Ning *et al*.^18^ have confirmed that mir-21 activates ERK/NF-κB by promoting degradation of the target gene Spry1. Hence, it can be inferred that mir-21 activates the NLRP3 inflammasome via the ERK/NF-κB pathway by targeting Spry1 in lung fibroblasts.

Previous studies by our group have demonstrated that the angiotensin-converting enzyme 2 (ACE2)/Ang(1–7)/Mas axis, which counteracts the activity of the ACE/AngII/AT1R axis, has protective effects against pulmonary fibrosis^19^. Other recent studies have shown that AngII promotes mir-21 expression^20,21^, and 1 study found that Ang(1–7) reduces mir-21 expression in muscle^22^. Therefore, we hypothesized that ACE2/Ang(1–7) prevents AngII-induced pulmonary fibrosis by inhibiting the expression of mir-21, and that mir-21 mediates the inhibitory effect of ACE2/Ang(1–7) on AngII-induced-NLRP3 inflammasome activation via the ERK/NF-κB pathway by targeting Spry1 in lung fibroblasts.

In this study, we investigated whether ACE2/Ang(1–7) antagonizes AngII-mediated pathophysiological activation of processes that lead to pulmonary fibrosis both *in vitro* and *in vivo*. We demonstrated that AngII-induced mir-21 activated the NLRP3 inflammasome in lung fibroblasts by targeting Spry1, resulting in apoptosis resistance and collagen synthesis. In contrast, ACE2/Ang(1–7) prevented AngII-induced collagen synthesis in lung fibroblasts and BLM-induced pulmonary fibrosis by inhibiting the expression of mir-21.

## Results

### Mir-21 inhibited apoptosis, promoted collagen synthesis, and activated the NLRP3 inflamasome via the Spry1/ERK/NF-κB pathway in lung fibroblasts

**Figure 1:**
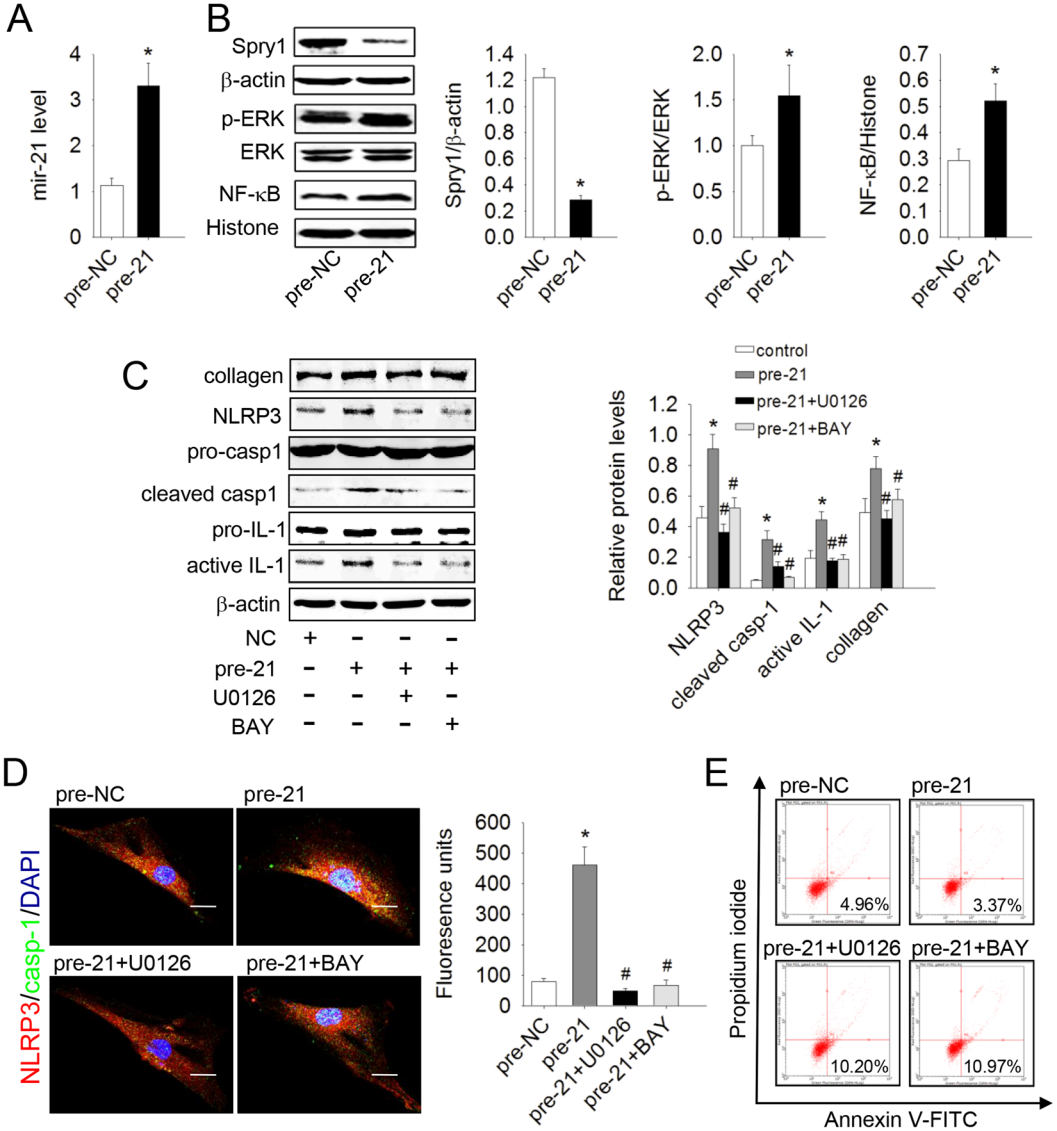
Over-expression of mir-21 inhibited apoptosis and promoted collagen synthesis by activating the ERK/NF-κB /NLRP3 inflammasome pathway via targeting Spry1 in lung fibroblasts. Primary lung fibroblasts were transfected with control miRNA precursors (pre-NC) or mir-21 precursors (pre-21) for 48 h and then treated with U0126 (10^−5^ mol/L) or BAY 117082 (10^−5^ mol/L). (**A**) The expression of mir-21 in lung fibroblasts was determined by qRT-PCR. (**B**) The protein levels of Spry1, p-ERK, and NF-κB were analyzed by western blot. (**C**) The protein levels of NLRP3, pro-caspase-1 (pro-casp-1), cleaved caspase-1 (cleaved casp-1), Pro-IL-1β, active IL-1β, and a-collagen I were analyzed by western blot. (**D**) Dual immunofluorescence of NLRP3 (red) and caspase-1 (green). Nuclei were stained with DAPI (blue). The yellow Fluorescence units were measured, which represent the co-location of NLRP3 and casp-1. The images are representative of 3 separate experiments. (**E**) Apoptosis of lung fibroblasts was evaluated by flow cytometry. Scale bar = 20 μm. Data are the means ± SD from 3 independent experiments. *P < 0.05 versus NC; ^#^ *P* < 0.05 versus pre-21.

Mir-21 has been shown to play an important role in fibrotic diseases. Up-regulated mir-21 enhances transcription of fibronectin (FN) and α-smooth muscle actin (α-SMA), but gene silencing of mir-21 inhibits collagen deposition and myofibroblast differentiation^4^. To study the role of mir-21 in lung fibroblasts, we enhanced the mir-21 level by transfection with mir-21 precursors (pre-21), and qRT-PCR analysis showed that mir-21 was increased significantly compared with pre-NC (control miRNA precursors) group (Fig. 1A). Overexpression of mir-21 inhibited apoptosis and up-regulated a-collagen I synthesis in lung fibroblasts (Fig. 1C,E) accompanied by increased p-ERK- NLRP3 inflammasome complex (NLRP3, Pro-caspase-1), Pro-IL-1β, and IL-1β protein levels, but decreased Spry1 protein levels (Fig. 1B,C). Conversely, U0126 and BAY117082 inhibited NF-κB translocation, the protein levels of Pro-IL-1β, IL-1β, NLRP3 inflammasome complex, and caspase-1 induced by pre-mir-21 (Fig. 1B,C,E), as well as apoptosis resistance and a-collagen I synthesis. Immunofluorescence staining showed that mir-21 induced co-localization of NLRP3 and caspase-1 in cytoplasm, which could be abolished by U0126 and BAY 117082 (Fig. 1D). These data indicate that mir-21 inhibited apoptosis and promoted a-collagen I synthesis by activating the NLRP3 inflammasome via the Spry1/ERK/NF-κB pathway in lung fibroblasts.

### AngII‒up-regulated mir-21 led to apoptosis resistance and collagen synthesis in lung fibroblasts

**Figure 2:**
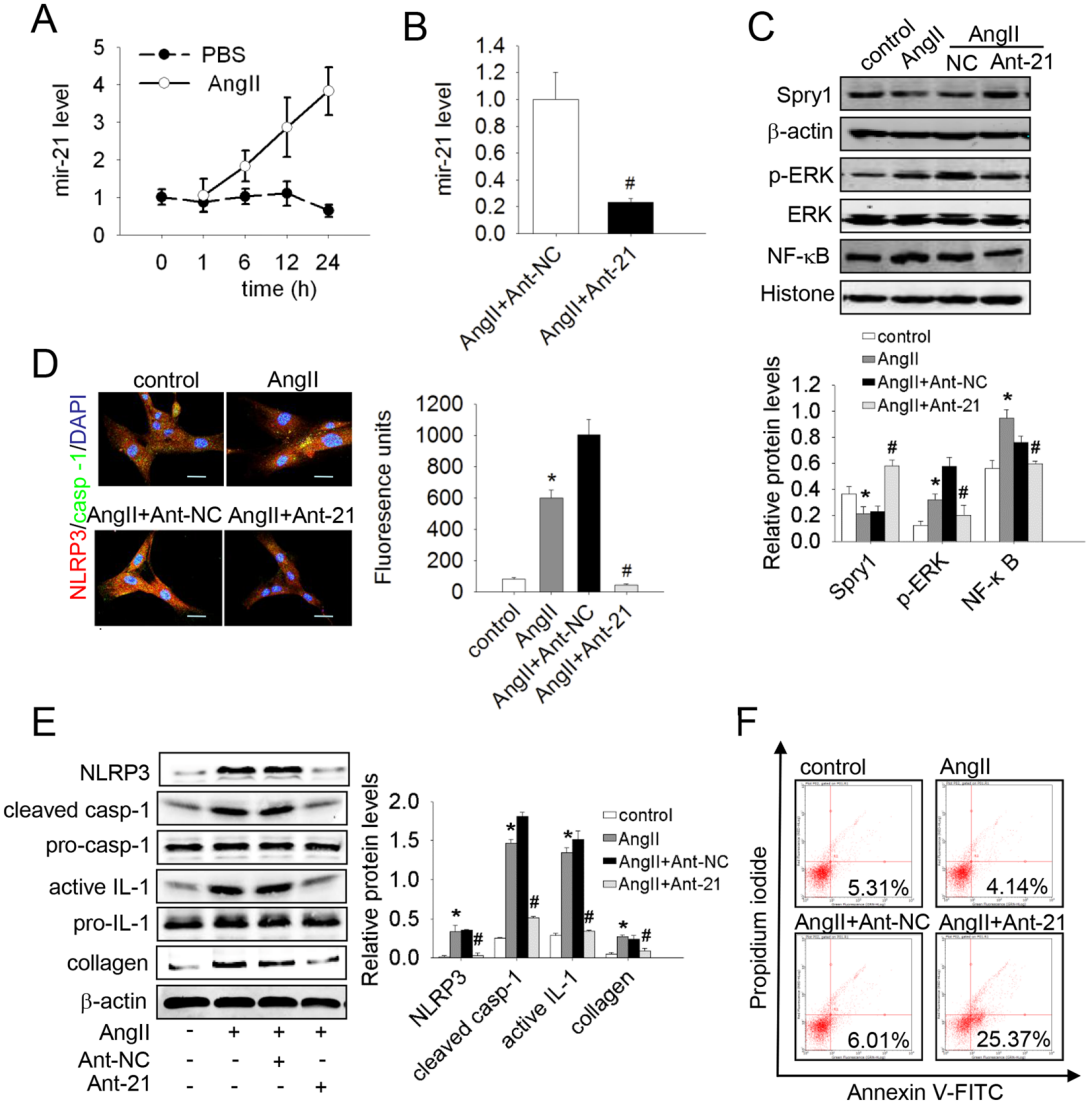
Inhibition of mir-21 suppressed AngII-induced apoptosis resistance, collagen synthesis, and the Spry1/ERK/NF-κB /NLRP3 inflammasome pathway in lung fibroblasts. (**A**) Primary lung fibroblasts were treated with AngII (10^−7^ mol/L) at different times. The mir-21 levels were detected by qRT-PCR. (**B-F**) Lung fibroblasts were transfected with anti-mir-NC (Ant-NC) or anti-mir-21 (Ant-21), and after 48 h the cells were treated with AngII (10^−7^ mol/L) for an additional 24 h. (**B**) The mir-21 levels were detected by qRT-PCR. (**C**) The protein levels of Spry1, p-ERK, and NF-κB were analyzed by western blot. (**D**) Dual immunofluorescence for NLRP3 (red) and caspase-1 (green). Nuclei were stained with DAPI (blue). The yellow Fluorescence units were measured, which represent the co-location of NLRP3 and casp-1. Images are representative of 3 separate experiments. (**E**) The protein levels of NLRP3, Pro-casp-1, cleaved casp-1, Pro-IL-1β, active IL-1β and a-collagen I were analyzed by western blot. (**F**) Apoptosis of cells was evaluated by flow cytometry. Scale bar = 40 μm. Data are the means ± SD from 3 independent experiments. *P < 0.05 versus control; ^#^ *P* < 0.05 versus AngII or AngII + Anti-NC.

It is now becoming apparent that AngII is involved in lung fibrogenesis^23^. However, the precise mechanism of this effect needs to be further explored. In 2012, Adam *et al*.^24^ reported that AngII up-regulates the expression of mir-21 in cardiac fibroblasts. To test whether AngII promotes mir-21 expression in lung fibroblasts, the cells were treated with AngII (10^−7^ mmol/L) at different time points, and the expression of mir-21 was determined. As shown in Fig. 2A, *the* mir-21 level increased significantly in a time-dependent manner after treatment with AngII. Consistent with our previous findings, AngII treatment inhibited apoptosis and promoted a-collagen I synthesis in lung fibroblasts (Fig. 2E,F). Furthermore, protein levels of ERK/NF-κB and the NLRP3 inflammasome were increased, but Spry1 was decreased after AngII treatment (Fig. 2C,E). We therefore explored whether the effects of AngII are mediated by mir-21. Mir-21 antisense probes were used to reduce mir-21 expression level in AngII-treated cells (Fig. 2B). AngII-induced changes of protein levels of the Spry1/ERK/NF-κB/NLRP3 inflammasome pathway and a-collagen I were restored by mir-21 antisense probes (Fig. 2C,E). Similarly, knocking down mir-21 inhibited the effect on apoptosis resistance of AngII (Fig. 2F). Immunofluorescent staining of NLRP3 and caspase-1 produced the same result (Fig. 2D). Thus, mir-21 mediated AngII-induced apoptosis resistance and a-collagen I synthesis in lung fibroblasts.

### Mir-21 mediated the inhibitory effect of ACE2/Ang(1–7) on AngII-induced apoptosis resistance and a-collagen I synthesis in lung fibroblasts

**Figure 3:**
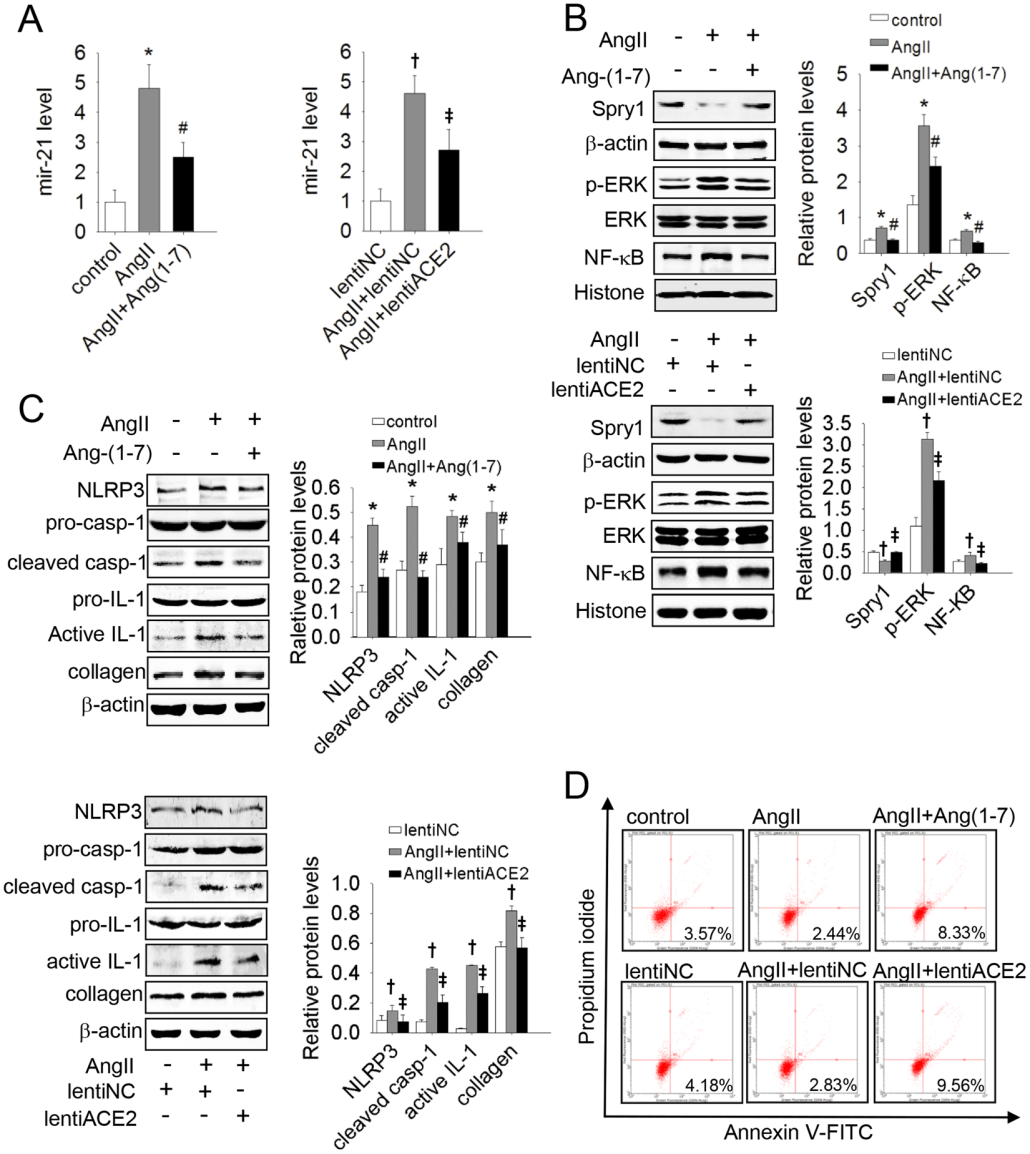
ACE2/Ang(1–7) inhibited AngII-induced apoptosis resistance and collagen synthesis by inhibiting the mir-21-mediated Spry1/ERK/NF-κB NLRP3 inflammasome pathway. Primary lung fibroblasts were infected with lentiACE2 or pretreated with Ang(1–7) and then exposed to AngII. (**A**) The mir-21 levels were detected by qRT-PCR. (**B**) The protein levels of Spry1 p-ERK and NF-κB were analyzed by western blot. (**C**) The protein levels of NLRP3, pro-casp-1, cleaved casp-1, pro-IL-1β, and active IL-1β were analyzed by western blot. (**D**) Apoptosis of cells was evaluated by flow cytometry. Data are the means ± SD from 3 independent experiments. *P < 0.05 versus control; ^#^ *P* < 0.05 versus AngII; ^†^ *P* < 0.05 versus lentiNC; ^‡^ *P* < 0.05 versus AngII + lentiNC.

In recent years, the protective effects of ACE2/Ang(1–7) during progression of IPF have been increasingly investigated^25,26^, especially with regard to the actions of ACE2/Ang(1–7) in counteracting the pro-fibrotic effects of AngII. We therefore investigated whether ACE2 and Ang(1–7) interfere with the effects of AngII on mir-21 expression. As shown in Fig. 3A, ACE2/Ang(1–7) markedly decreased the expression of mir-21 induced by AngII. The Spry1/ERK/NF-κB/NLRP3 inflammasome pathway was activated and a-collagen I synthesis was markedly increased in response to AngII treatment in lung fibroblasts, along with decreased apoptosis. This response was abolished by ACE2 and Ang(1–7) [Fig. 3B–D]. These *in vitro* results suggest that mir-21 mediated the inhibitory effect of ACE2/Ang(1–7) on AngII-induced apoptosis resistance and a-collagen I synthesis in lung fibroblasts.

### ACE2/Ang(1–7) failed to inhibit NLRP3 inflammasome activation and a-collagen I induced by mir-21 over-expression

**Figure 4:**
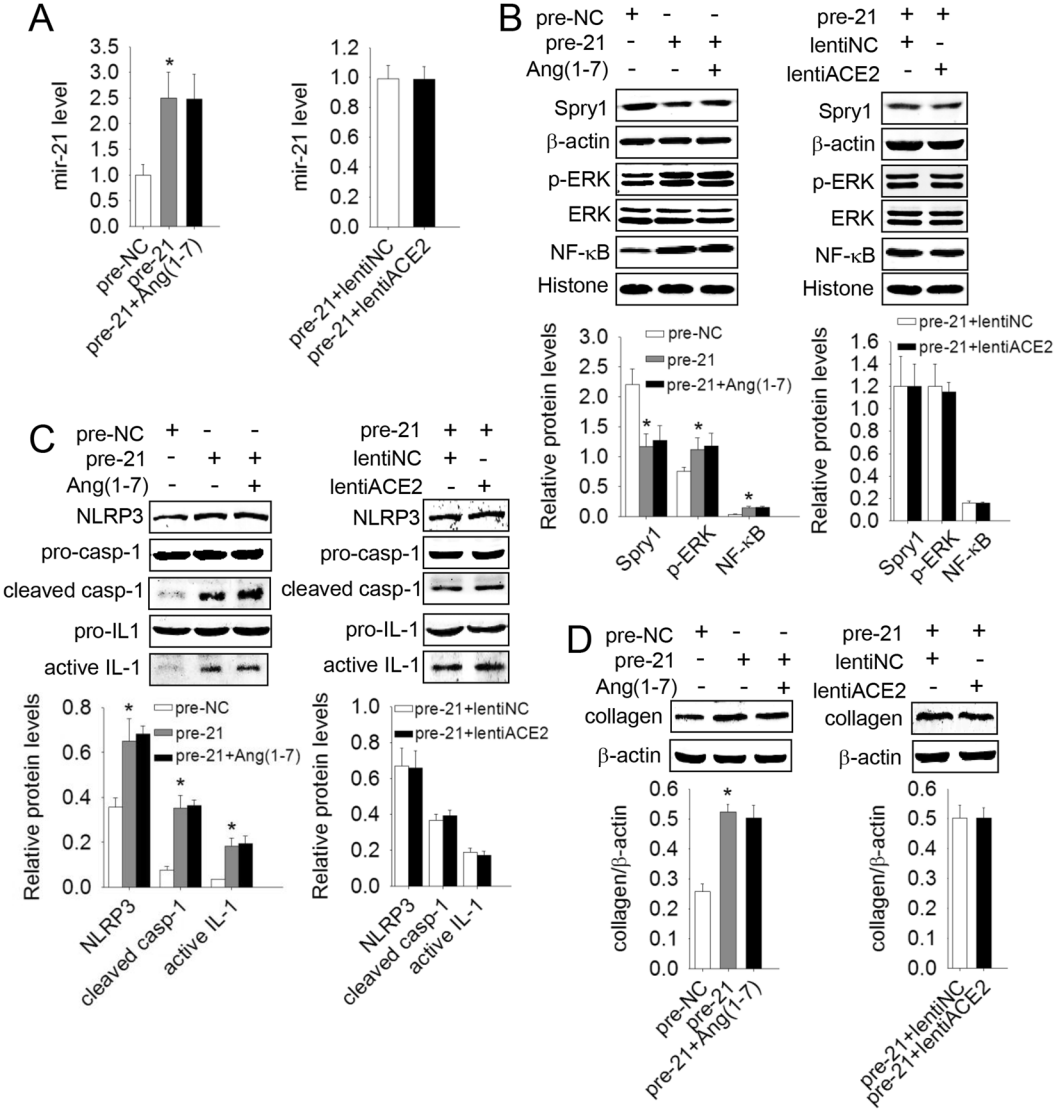
ACE2/Ang-(1–7) had no influence on the downstream pathway of mir-21. Primary lung fibroblasts were transfected with mir-21 precusors (pre-21) after the cells had been transfected with lentiACE2 or pretreated with Ang(1–7) for 24 h. (**A**) The mir-21 levels were detected by qRT-PCR. (**B**–**D**) The protein levels of Spry1, p-ERK, NF-κB, NLRP3, Pro-casp-1, cleaved casp-1, Pro-IL-1β, active IL-1β and a-collagen I were analyzed by western blot. Data are the means ± SD from 3 independent experiments. *P < 0.05 versus pre-NC.

In investigating the links of ACE2 /Ang(1–7) and mir-21 in pulmonary fibrosis, we found that the protein levels of ERK/NF-κB, the NLRP3 inflammasome, and collagen were increased, but Spry1 was decreased by over-expression of mir-21, and these effects could not be reversed by ACE2/Ang(1–7) [Fig. 4A,B,C,D]. Neither ACE2 nor Ang(1–7) influenced the function or the downstream molecular signaling of mir-21, but they did influence its expression.

### Mir-21 mediated the contravariant response of ACE2/Ang(1–7) and AngII in BLM-induced lung fibrosis

**Figure 5:**
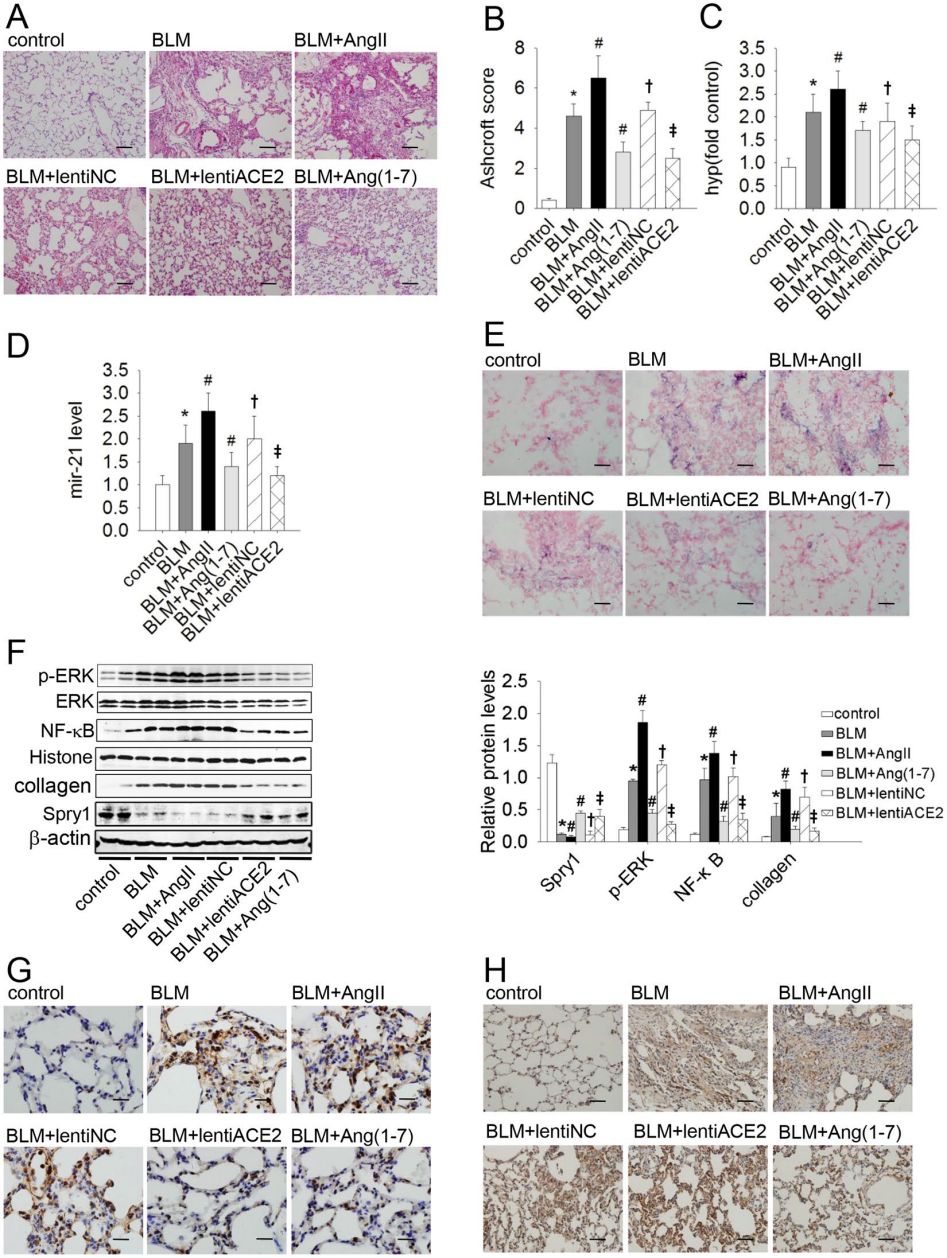
Bleomycin (BLM)-induced pulmonary fibrosis was attenuated by ACE2/Ang(1–7) and exacerbated by AngII via different effects on the mir-21-mediated Spry1/ERK/NF-κB/NLRP3 pathway. Representative microphotographs of lung sections from controls and the BLM, BLM + AngII, BLM + Ang(1–7), BLM + lentiACE2, and BLM + lentiNC groups [n = 12 rats per group] stained with H&E are shown (original magnification × 200; scale bar = 100 μm). (**A**,**B**) Morphological changes in fibrotic lungs were quantified by the Ashcroft score. (**C**) The hydroxyproline content of lungs in the different groups. (**D**) The expression of mir-21 in lungs was determined by qRT-PCR. (**E**) *In situ* hybridization (ISH) staining was performed to determine the localization and expression of mir-21 (original magnification × 200; scale bar = 50 μm). (**F**) The protein levels of Spry1, p-ERK, NF-κB, and collagen in lung tissues were analyzed by western blot. (**G**,**H**) Immunohistochemical staining was performed to determine the localization and expression of NF-κB (G: original magnification × 400; scale bar = 50 μm) and NLRP3 (H: original magnification × 200; scale bar = 100 μm) proteins. The data are presented as means ± SD. *P < 0.05 versus control; ^#^ *P* < 0.05 versus BLM; ^†^ *P* < 0.05 versus BLM; ^‡^ *P* < 0.05 versus BLM + lentiNC.

Compared with control rats, BLM treatment alone was associated with higher Ashcroft scores, higher levels of hydroxyproline, and severe fibrosis with marked mononuclear infiltration and thickened alveolar septa throughout the lung parenchyma. Infusion of Ang(1–7) or over-expression of ACE2 was associated with markedly lower scores and a reduced degree of fibrotic lesions. However, the pro-fibrotic effect was more evident in AngII-treated rats (Fig. 5A,B,C). These data suggest that AngII exacerbated and ACE2/Ang(1–7) attenuated BLM-induced pulmonary fibrosis in rats.

We then investigated the role of mir-21 in this rat model. The mir-21 level was markedly enhanced in the BLM group compared with the control group. Although the mir-21 level was markedly reduced by infusion of Ang(1–7) or over-expression of ACE2, it was augmented by Ang II treatment (Fig. 5D,E). Moreover, protein levels of ERK/NF-κB/NLRP3 and collagen were increased, but the protein level of Spry1 was decreased in BLM-induced pulmonary fibrosis. This response was abolished by ACE2 or Ang(1–7) treatment and exacerbated by Ang II (Fig. 5F,G,H). Similarly, ACE2 or Ang(1–7) reduced the a-collagen I protein levels induced by BLM, which were aggravated by AngII treatment (Fig. 5F). Together with the *in vitro* results, we concluded that ACE2/Ang(1–7) attenuated and AngII exacerbated BLM-induced lung fibrosis by modulation of mir-21.

**Figure 6:**
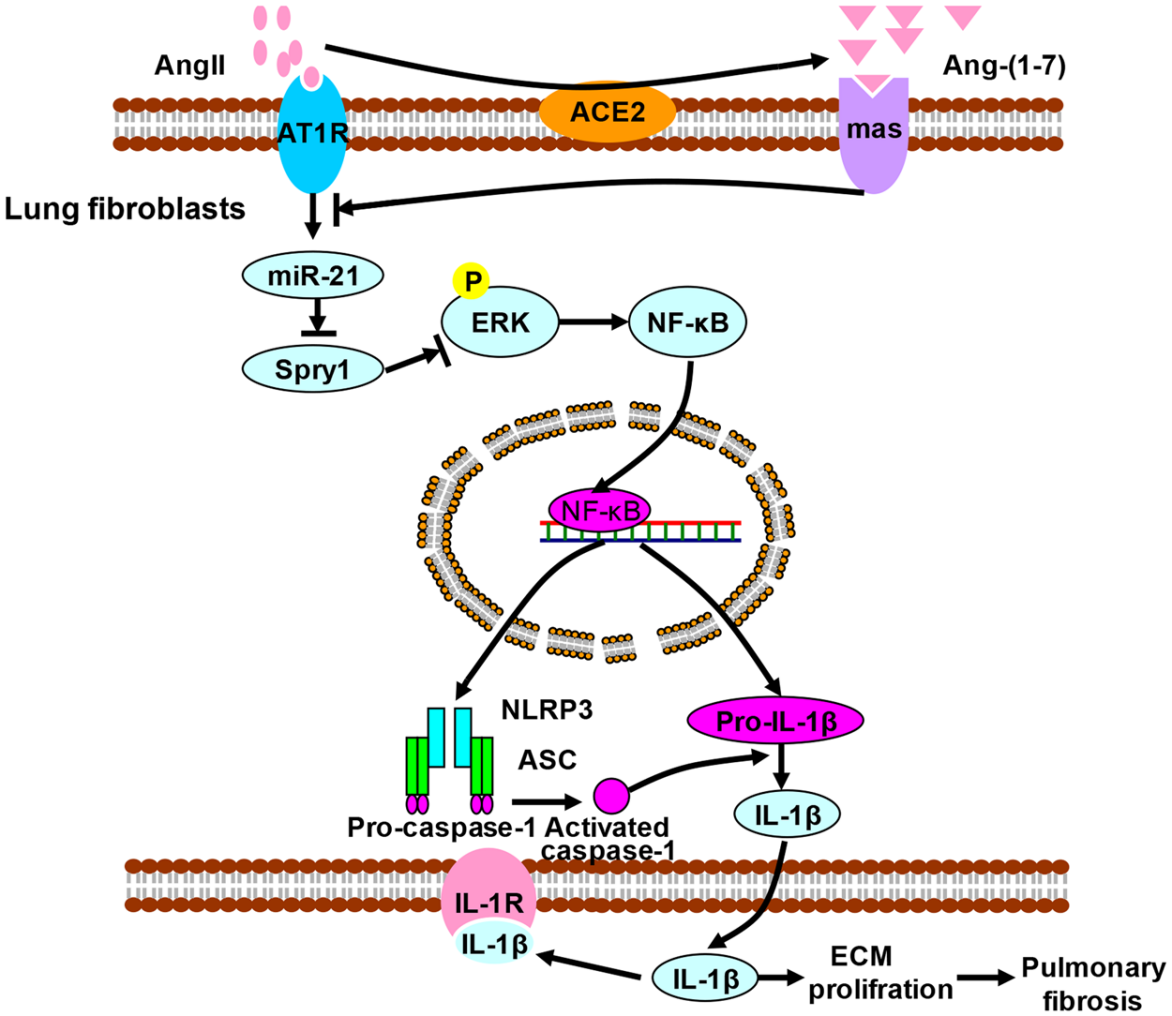
Schematic working hypothesis. It has been demonstrated local RAAS is activated in lung tissue of fibrosis. AngII binds with AT1R in lung fibroblasts to enhance the expression level of mir-21, the recognized pro-fibrotic factor. Mir-21 promotes ERK phosphorylation and NF-κB nuclei translocation by targeting Spry1 degradation, following NLRP3 inflammasome activation. The released proinflammatory factors (Caspase-1/IL-1) by the inflammasome pathway lead to a-colllagen I synthesis and apoptosis resisitance. ACE2 degrades AngII to produce Ang(1–7), which reverses the AngII’s profibrotic effect via downregulating mir-21. As a result, mir-21 and NLRP3 inflammasome play a key in local RAAS imbalance induced lung fibrosis.

Taken together, these data demonstrate that mir-21 mediates the inhibition of ACE2/Ang (1–7) on angiotensin II induced-NLRP3 inflammasome activation via ERK/NF-κB pathway by targeting Spry1 in lung fibroblasts (Fig. 6).

## Discussion

In this study, we have demonstrated that AngII-upregulated mir-21 activates the NLRP3 inflammasome by targeting Spry1, which promoted lung fibroblast collagen synthesis and exacerbated BLM-induced fibrosis. However, ACE2/Ang(1–7) antagonized the pro-fibrotic effect of AngII by down-regulating mir-21 expression. The study’s principal findings are that: (1) mir-21 mediates collagen synthesis by activating the ERK/NF-κB/NLRP3 inflammsome pathway via targeting Spry1 in lung fibroblasts; (2) mir-21 is up-regulated in primary lung fibroblasts treated with AngII and in BLM-induced pulmonary fibrosis; and (3) mir-21 mediates the inhibitory effect of ACE2/Ang(1–7) on AngII-induced NLRP3 inflammasome activation by targeting Spry1 in lung fibroblasts.

Mir-21 is recognized as an oncomiR due to its activity on cellar proliferation, differentiation, and apoptosis. It has been extensively researched and serves as a potential biomarker for diagnosing cancer and determining its prognosis^27^. Increasing additional roles^28–30^ in other diseases suggest that elevated mir-21 expression may represent a common feature of pathological progression, including fibrotic diseases. It has been reported that mir-21 levels are up-regulated in fibrosis of the kidneys^31^, heart^32^, liver^9^, and lungs^7^, and that targeting mir-21 therapeutically may have the ability to prevent development of organ fibrosis^4^. Furthermore, recent studies have shown that mir-21 is involved in the promotion of fibrogenic activation of fibroblasts in different organs^2^. Consistent with these results, we found that mir-21 l was markedly up-regulated in the lung of BLM-treated rats *in vivo*, and that over-expression of mir-21 inhibited lung fibroblast apoptosis and promoted collagen synthesis *in vitro*. As it has also been shown that the pro-fibrotic factors transforming growth factor (TGF)-β^33^ and platelet-derived growth factor (PDGF)^4^ promote fibrogenesis by up-regulating mir-21, we investigated whether other pro-fibrotic factors can activate mir-21. AngII, the principal effector peptide of the vasoconstrictor arm of the renin-angiotensin system (RAS), has a key role in the initiation and perpetuation of inflammation and fibrosis in experimental lung fibrosis^34^, and it is now becoming apparent that mir-21 is transcriptionally activated in cardiac fibrosis related to AngII^32^. Adam *et al*.^24^ have also reported that AngII up-regulates the expression of mir-21 in cardiac fibroblasts, resulting in their activation and atrial fibrosis. Thus, we propose that the pro-fibrotic effect of AngII may be associated with its augmentation of mir-21. Consistent with previous findings, we found that mir-21 levels were significantly increased in lung fibroblasts treated with AngII. Moreover, the effects of AngII in inhibiting apoptosis and promoting collagen synthesis can be reversed by silencing mir-21 in lung fibroblasts. In BLM-treated rats, constant infusion of exogenous AngII significantly up-regulated the expression of mir-21 and aggravated lung fibrosis. Hence, AngII-induced mir-21 promoted collagen synthesis in lung fibroblasts and exacerbated BLM-induced lung fibrosis.

Although the pro-fibrotic effect of mir-21 is clear, the precise molecular mechanism by which it exerts this effect needs to be elucidated. A number of signaling pathways have been identified as being involved in the fibrogenensis mediated by mir-21 in different organs, including PTEN/Akt, NF-κB, ERK, TGF-β1/Smad, and IL-13/Smad signaling pathways^35^. Ning *et al*.^18^ discovered that Spry1, an important target gene of mir-21, can inhibit the ERK/NF-κB pathway, which is involved in liver fibrosis^18^. In this study, we have shown that mir-21 over-expression or AngII-induced mir-21 significantly increased p-ERK and NF-κB nucleoprotein levels, but decreased the Spry1 protein level in lung fibroblasts. Moreover, mir-21 silencing inhibited the Spry1/ERK/ NF-κB pathway activation. Collectively, we demonstrated that AngII-induced mir-21 mediated ERK/NF-κB pathway activation by targeting Spry1 in lung fibroblasts. NF-κB is a nuclear transcription factor that regulates the expression of a large number of genes, including the NLRP3 inflammasome.

Recent studies have elucidated the important role of the NLRP3 inflammasome in the development of fibrosis in various organs, including the heart^36^, kidneys^37^, liver^10^, and lungs^38^. Artlett & Thacker^39^ consider that activation of the NLRP3 inflammasome is a common thread linking divergent fibrogenic diseases. Furthemore, our previous studies have demonstrated NLRP3 inflammasome mediated AngII-induced pulmonary fibrosis in animal models. In accordance with these results, we found the NLRP3 inflammasome/IL-1β secretion axis was activated by AngII or mir-21 over-expression in lung fibroblasts, and that this can be reversed by anti-mir-21 probes. Thus, mir-21 has a potential role in activation of the NLRP3 inflammasome/IL-1β secretion axis. However, how mir-21 exerts this effect remains to be elucidated.

In this study, overexpression of mir-21 promoted ERK phosphorylation and NF-κB nuclei translocation by inhibiting the Spry1 gene. By inhibiting ERK and NF-κB, U0126 and BAY 117082 inhibited mir-21 over-expression-induced activation of the NLRP3 inflammasome complex. Thus, mir-21 activated the NLRP3 inflammasome /IL-1β secretion axis via the ERK/NF-κB pathway by targeting Spry1.

The ACE2/Ang(1–7)/Mas pathway is involved in many physiological and pathophysiological processes in several systems and organs, notably by opposing the detrimental effects of inappropriate over-activation of the ACE/AngII/AT1R axis^40^. Li *et al*.^41^ consider that Ang(1–7) protects against experimental lung fibrosis by limiting the local tissue accumulation of AngII that occurs in response to BLM-induced lung injury, which is consistent with our previous studies. However, the precise molecular mechanism of the anti-fibrotic effect of ACE2/Ang(1–7) remains to be elucidated. We found that the effect of AngII-induced mir-21 was abolished by ACE2/Ang(1–7) in lung fibroblasts *in vitro*, and that exogenous Ang(1–7) infusion or lentiACE2 intratracheal instillation significantly decreased mir-21 expression and attenuated lung fibrosis induced by BLM *in vivo*. Furthermore, in primary lung fibroblasts, ACE2/Ang(1–7) suppressed AngII-induced ERK phosphorylation, NF-κB nuclei translocation, and degradation of the targeting gene Spry1, and blocked AngII-induced activation of the NLRP3 inflammasome. In exploring whether these responses of ACE2/Ang1–7 were mediated by mir-21, we found that activation of the Spry1/ERK/ NF-κB pathway and the NLRP3 inflammasome/IL-1β secretion axis by over-expression of mir-21 could not be reversed by ACE2/Ang(1–7). Overall, these data suggest that mir-21 mediates the inhibitory effect of ACE2/Ang (1–7) on AngII-induced activation of the NLRP3 inflammasome by targeting Spry1 in lung fibroblasts.

Excessive accumulation of mesenchymal cells, especially fibroblasts, is a feature of pulmonary fibrosis, and fibroblasts from fibrotic lungs are resistant to a variety of apoptotic stimuli^42^. Meng *et al*.^43^ found that AngII activates MAPK/NF-κB and promotes apoptosis resistance, resulting in accumulation of fibroblasts and augmented pulmonary fibrosis. Similarly, mir-21 has been shown to be involved in apoptosis resistance in a variety of disease states^29,30,44,45^. In accordance with these results, we found that AngII-induced mir-21 inhibited fibroblast apoptosis by the Spry1/ERK/ NF-κB pathway by targeting Spry1 degradation. Furthermore, ACE2/Ang(1–7) reversed the anti-apoptosis effect of AngII by down-regulating mir-21. However, the mechanism of this anti-apoptotic effect needs to be further elucidated.

*In conclusion*, our study has demonstrated that AngII induction of mir-21 is a crucial factor that mediates pulmonary fibrosis by activating the NLRP3 inflammasome/IL-1β secretion axis via the Spry1/ERK/NF-κB pathway. Exogenous ACE2/Ang(1–7) over-expression protected against BLM-induced pulmonary fibrosis by down-regulating mir-21. These findings suggest a critical role of mir-21 in both the pro-fibrotic effect of AngII and the anti-fibrotic effect of ACE2/ Ang(1–7) in BLM-induced pulmonary fibrosis. Consequently, down-regulation of mir-21 may be a promising strategy for the prevention and treatment of pulmonary fibrosis.

## Materials and Methods

### Materials

AngII, Ang(1–7), U0126 (a specific ERK1/2 inhibitor) and BAY117082 (an IκK inhibitor) were purchased from Sigma-Aldrich (St. Louis, Missouri, USA). Bleomycin (BLM) was purchased from Nippon Kayaku (Tokyo, Japan). Alzzet osmotic pumps (models 2004 and 2ML4) were purchased from Durect Corporation (Cupertino, CA, USA). Other reagents are described below.

### Animals

Male Wistar rats weighing 200–300 g were provided by the Central Animal Care Facility of Southern Medical University (Permission No. SCXK2009–015). All experimental procedures on rats were approved by the Committee on the Ethics of Animal Experiments of Southern Medical University and were performded in accordance with the World Medical Association’s Declaration of Helsinki. All animals were housed (12 h light/dark; temperature 22 °C–24 °C) and given food and water ad libitum in the Animal Experiment Center of Nanfang Hospital. The protocol for animal use in the study was approved by the Ethics of Animal Experiments Committee at Southern Medical University.

### Animal treatment regimens

A model of pulmonary fibrosis was established by intratracheal instillation of BLM. 92 male Wistar rats were randomly divided into 6 groups of 12 rats each: control, BLM, BLM + AngII, BLM + Ang(1–7), BLM + lentiACE2 (lentivirus- mediated ACE2), and BLM + lentiNC (lenti-empty virus) groups. To down-regulate the expression of ACE2, rats in the BLM + lentiACE2 group received 3 ×10^8^ TU lentivirus by intratracheal instillation under pentobarbital anesthesia. Those in the BLM + lentiNC group received an equal volume of lentiNC. Two weeks after lentiviral treatment, all of the rats received a single intratracheal instillation of 200 μL of sterile saline while under pentobarbital anesthesia. The 5 BLM groups then received sterile saline that contained 5 mg/kg of BLM sulfate. While the animals were under anesthesia, micro-osmotic pumps were subcutaneously implanted to permit 28 days of continuous infusion with AngII or Ang(1–7) at a rate of 25 μg•kg^−1^•h^−1^. The rats were sacrificed 4 weeks after BLM treatment, and lung samples were collected.

### Isolation of primary lung fibroblasts

Normal rat primary fibroblasts from male Wistar rats were prepared and cultured as previously described^19^. The cell were cultured in Dulbecco’s Modified Eagle’s medium (DMEM) supplemented with 15% fetal bovine serum (Gibco). All experiments were performed with 3–5 passages.

### Production of lentiACE2 viral particles

Lentiviral particles containing enhanced green fluorescent protein (pGC-FU-GFP, lenti-GFP/lentiNC) or human ACE2 (pGC-FU-ACE2-GFP, lentiACE2) were prepared. Viral medium containing lenti-GFP or lentiACE2 was then collected, concentrated and titered. The concentration of viral particles was determined using quantitative real-time polymerase chain reaction (qRT-PCR) technology. The efficacy of lentiACE2 in producing active ACE2 enzymes has been previously established.

### Histological analysis and immunochemical assessment

The right lung was fixed using an intratracheal instillation of 4% paraformaldehyde, embedded in paraffin, and cut into 5 μm thick sections. Hematoxylin and eosin (H&E) staining was used to identify alveolitis and fibrosis. The severity of pathological changes was scored according to the Ashcroft scale, and the presence of collagen was assessed by analyzing the stained area as a percentage of the total area.

For immunohistochemical (IHC) analysis, sections were stained with anti-NF-κB and anti-NLRP3 antibodies. After incubation with streptavidin peroxidase-conjugated secondary antibody, peroxidase conjugates were visualized with diaminobenzidine and observed under a light microscope.

### Hydroxyproline assay

The lung tissue was hydrolyzed, followed by derivation using the HYP analysis kit (Sigma). The collagen content was measured and analyzed by ultraviolet spectrophotometry, using the procedure recommended by the manufacturer.

### *In situ* hybridization (ISH)

Lung sections were treated with an acetylation solution and proteinase K. The sections were then blocked with hybridization solution and incubated with digoxigenin-conjugated mir-21 probes (Exiqon, Denmark). Subsequently, the sections were incubated with the HRP-conjugated anti-digoxigenin antibody (Roche, Shanghai, China). Finally, the sections were treated with NBT/BCIP (Roche, Shanghai, China). Light blue cytoplasmic staining indicates a positive test.

### Immunofluorescence analysis

Primary lung fibroblasts grown on coverslips were fixed in 4% paraformaldehyde and then incubated with anti-NLRP3 antibody and anti-caspase-1 antibody, followed by Cy3-conjugated anti-mouse or FITC (fluorescein isothiocyanate)-conjugated anti-rabbit secondary antibodies. The nuclei were stained with DAPI (4′,6-diamidino-2-phenylindole). Fluorescence was visualized using an Olympus FV10i-W confocal microscope. Controls with no primary antibody showed no fluorescence labeling. Single label controls were performed in the double-labeling experiments.

### Western blot analysis

Lung tissue was lysed with radioimmunoprecipitation assay (RIPA) buffer (Beyotime, China) to extract the total protein. A bicinchoninic acid (BCA) protein assay was used to determine the protein concentration. The lysates were mixed with sodium dodecyl sulfate (SDS) buffer and denatured at 100 °C for 5 min. Equal concentrations of proteins were then separated on sodium dodecyl sulfate polyacrylamide gel electrophoresis (SDS-PAGE) gels and transferred to polyvinylidene difluoride (PVDF) membranes. The membranes were probed with primary antibodies at 4 °C overnight, including anti-collagen I, anti-Spry1, anti-p-ERK1/2, anti-ERK1/2, anti-NF-κB, anti-β-actin, and anti-histone (1:1000; Cell Signaling Technology, Massachusetts, USA), and then incubated with anti-rabbit near-infrared secondary antibody (1:15000, Li-Cor, USA) for 1 h. The membrane was exposed to Odyssey^®^ CLx Imager, and Odyssey Software was used for capturing images and the data analysis. The experiment was repeated 3 times for consistency.

### Quantitative real-time PCR analysis (qRT-PCR)

The total RNA of cultured cells and lung tissue was isolated by using Trizol reagent (Invitrogen, Life Technologies, NY, USA). The All-in-One mir-21q-RT-PCR kit (GeneCopoeia, Guangzhou, China) was used according to the manufacturer’s instructions. qRT-PCR was performed using the ABI 7500 Real-Time PCR System (Applied Biosystems). The universal primer of mir-21, UAGCUUAUCAGACUGAUGUUGA, was used during reverse transcription, and the results were normalized to U6.

### Flow cytometry

Cell apoptosis was examined with flow cytometry analysis. Briefly, the cells were collected and washed twice with phosphate-buffered saline (PBS), fixed in 2% paraformaldehyde for 30 minutes, and permeabilized using 0.1% Triton-X for 30 minutes. They were then stained with FITC-conjugated annexin V and propidium iodide (PI) using the Apoptosis Detection Kit (Invitrogen, USA) according to the manufacturer’s instructions. The apoptotic rate was measured with flow cytometry (FACS Calibur™, BD Biosciences, Franklin Lanes, NJ, USA), and the data were analyzed using CellQuest software. Each experiment was performed in triplicate.

### Statistical analysis

All data were presented as means ± standard deviation (SD). Significant differences were evaluated by analysis of variance (ANOVA) with the least significant difference (LSD) for multiple comparisons. Differences were considered statistically significant at a *P*-value of <0.05. All data were analyzed using SPSS^®^ software, version 13.0 (SPSS Inc, Chicago, IL, USA).

### Date availability

All data generated or analysed during this study are included in this published article.
