# Author Correction: Mir-21 Mediates the Inhibitory Effect of Ang (1–7) on AngII-induced NLRP3 Inflammasome Activation by Targeting Spry1 in lung fibroblasts

**DOI:** 10.1038/s41598-020-78308-z

**Published:** 2020-12-08

**Authors:** Na-Na Sun, Chang- Hui Yu, Miao-Xia Pan, Yue Zhang, Bo-Jun Zheng, Qian- Jie Yang, Ze -Mao Zheng, Ying Meng

**Affiliations:** Department of Respiratory Diseases, Nanfang Hospital, Southern Medical University, Guangzhou, China

Correction to: *Scientific Reports* 10.1038/s41598-017-13305-3, published online 30 October 2017

This Article contains an error in Figure 1C, where the representative blot for NLRP3 is incorrect.

The correct Figure 1 appears below.

**Figure 1:**
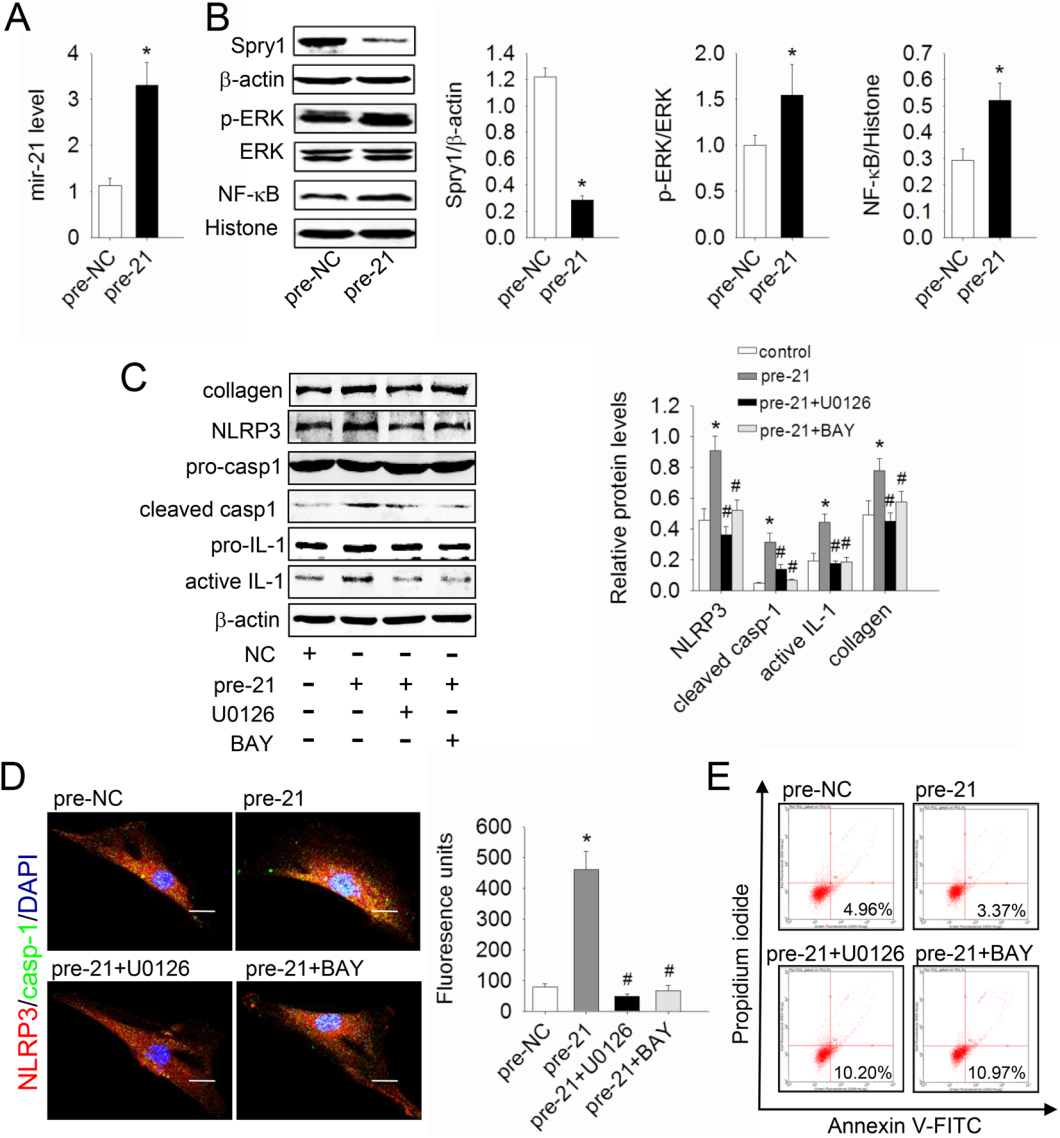
A correct version of the original Figure 1.

